# Contrasting Infection Strategies in Generalist and Specialist Wasp Parasitoids of Drosophila melanogaster

**DOI:** 10.1371/journal.ppat.0030158

**Published:** 2007-10-26

**Authors:** Todd A Schlenke, Jorge Morales, Shubha Govind, Andrew G Clark

**Affiliations:** 1 Department of Molecular Biology and Genetics, Cornell University, Ithaca, New York, United States of America; 2 Department of Biology, Emory University, Atlanta, Georgia, United States of America; 3 Biology Department, City College and the Graduate Center of The City University of New York, New York, New York, United States of America; Stanford University, United States of America

## Abstract

Although host–parasitoid interactions are becoming well characterized at the organismal and cellular levels, much remains to be understood of the molecular bases for the host immune response and the parasitoids' ability to defeat this immune response. Leptopilina boulardi and L. heterotoma, two closely related, highly infectious natural parasitoids of Drosophila melanogaster, appear to use very different infection strategies at the cellular level. Here, we further characterize cellular level differences in the infection characteristics of these two wasp species using newly derived, virulent inbred strains, and then use whole genome microarrays to compare the transcriptional response of *Drosophila* to each. While flies attacked by the *melanogaster* group specialist L. boulardi (strain Lb17) up-regulate numerous genes encoding proteolytic enzymes, components of the Toll and JAK/STAT pathways, and the melanization cascade as part of a combined cellular and humoral innate immune response, flies attacked by the generalist L. heterotoma (strain Lh14) do not appear to initiate an immune transcriptional response at the time points post-infection we assayed, perhaps due to the rapid venom-mediated lysis of host hemocytes (blood cells). Thus, the specialist parasitoid appears to invoke a full-blown immune response in the host, but suppresses and/or evades downstream components of this response. Given that activation of the host immune response likely depletes the energetic resources of the host, the specialist's infection strategy seems relatively disadvantageous. However, we uncover the mechanism for one potentially important fitness tradeoff of the generalist's highly immune suppressive infection strategy.

## Author Summary

The fruitfly Drosophila melanogaster has become a model system for the study of innate immunity, and parasitic wasps are one of the most obvious natural pathogens of *Drosophila*, making this a great system for studying interactions between the host immune system and pathogen virulence proteins. We have focused on two closely related wasp species, Leptopilina boulardi and L. heterotoma, that successfully parasitize D. melanogaster hosts in nature. Both wasps inject venom loaded with virus-like particles into their hosts to prevent host-mediated melanotic encapsulation and killing of their eggs. However, there are substantial differences in the effects of the venom from these two wasp species. L. heterotoma venom causes lysis of host hemocytes (blood cells) and prevents the host from mounting any substantial immune transcriptional response, while L. boulardi venom has a relatively weak and localized effect on host hemocyte survival and does not prevent immune response activation. Thus, these wasps allow us to compare the benefits and drawbacks of relatively immune suppressive versus relatively immune evasive parasite infection strategies in a natural system.

## Introduction

Parasitic wasps are exceedingly diverse, they often act as keystone species in natural ecosystems, and because of their ability to evade and/or suppress insect immune defenses they have become the most successful group of biological control agents [1]. Like many insects, *Drosophila* are regularly attacked by female parasitoid wasps, which use their ovipositors to inject eggs into *Drosophila* larvae (Video S1). If unchecked, wasp larvae hatch approximately 2 d post-infection and feed upon host tissues, eventually emerging from and killing the host pupae. In some natural populations, more than fifty percent of *Drosophila* larvae collected are infected, indicating that wasps can be a potent cause of mortality in immature flies [2,3]. However, once infected, *Drosophila* do not act as benign habitats for parasite growth.

*Drosophila* can mount a potent innate immune response against parasitic wasps and other pathogens. This immune response is often divided into two main components, the humoral response and the cellular response. The humoral response has been intensely studied for its role in combating bacterial and fungal infections, but may also be responsible for aspects of macroparasite killing. It is governed by the fat body, which controls release of immune active extracellular proteins such as antimicrobial peptides (AMPs) and complement-like proteins (e.g., Teps) into the hemolymph. The two major humoral immune response pathways operating in the fat body are the NF-κB pathways Toll and Imd, to which the JAK/STAT and JNK pathways appear to play complementary roles [4,5]. *Drosophila* responds to septic injury with bacteria and fungi by up-regulating many genes from the Toll and Imd pathways [6,7].

The *Drosophila* cellular response is mediated by the lymph gland (the hematopoietic organ) and the hemocytes, and is responsible for the phagocytosis of foreign cells and the hemocytic encapsulation of the larger macroparasites. In response to parasitoid wasps, hemocytes of resistant fly larvae are activated and migrate toward the wasp egg, and the lymph gland is stimulated to produce flattened lamellocytes that encapsulate both the egg and attached hemocytes [8]. Lysis of the inner layer of hemocytes results in deposition of dark melanin around the wasp egg, and cytotoxic free radicals produced within the melanotic capsule help kill the egg [9].

Several genetic pathways and individual genes have been implicated in, or have been directly shown to participate in (underlined text), the encapsulation response against macroparasites. These include genes involved in hematopoiesis and hemocyte survival (Toll pathway, JAK/STAT pathway, Ras pathway, Vegfr pathway, *srp*, *He*, *ytr*, *Tsp68C*, *brm*, *dom*, *mxc*, *Myb*, *Iswi*, *E(bx)*, *mod*, *CBP*…), hemocyte differentiation (Toll pathway, JAK/STAT pathway, Ras pathway, Notch pathway, *srp*, *lz*, *ush*, *cher*, *kn*, *gcm*, *gcm2*, *l(3)mbn*…), and melanization (phenoloxidase cascade) [10–25]. A handful of other genes also have identified roles in hemocyte cytoskeletal arrangements necessary for hemocyte activation, migration, and spreading (JNK pathway, Vegfr pathway, *He*, *Rac1*, *Rac2*, *Pi3K92E*…) [16,26–29]. Other genetic pathways, such as Imd and Wnt, may play complementary roles [25,30]. Finally, at least two whole-genome, gene expression (microarray) studies of *Drosophila*'s anti-parasite immune response resulted in a large assortment of candidate cellular immunity genes [31,32]. In particular, Wertheim et al. [32] identified 129 genes significantly differentially regulated in D. melanogaster after attack by a relatively avirulent strain of the parasitoid wasp Asobara tabida, including genes from biological function categories such as immune response, proteolysis, and development.

Despite counter-selection in their hosts, parasitic wasps have evolved numerous methods for defeating host immune responses, including strategies for passive immune evasion and for active immune suppression. For example, the *Drosophila* parasitic wasp A. tabida can passively evade the immune response by using “sticky” eggs that become embedded in host tissue and hidden from circulating hemocytes [33]. Host immune suppression by parasitic wasps is usually carried out by venom co-injected with the eggs, which often contains wasp-encoded viruses (e.g*.*, polydnaviruses) or virus-like particles (VLPs) [34]. Some polydnaviruses have been shown to block NF-κB signaling pathways, interfere with hemocyte spreading and adherence, and inhibit phenoloxidase (PO) activity (responsible for melanization) [35], and the nucleic acid-lacking VLPs of many *Drosophila* parasitic wasps may have similar effects.

Several wasp species are known to attack D. melanogaster larvae in nature, including the closely related Figitids L. heterotoma and *L. boulardi* [2,36]. L. heterotoma is distributed across the holarctic region, whereas L. boulardi is mainly known from Mediterranean and tropical climates, but they are sympatric for large portions of their species ranges [37]. Both these wasp species are remarkably adapted to successful parasitization of D. melanogaster, as strains relatively avirulent on D. melanogaster are rarely collected in the field [38]. However, at the cellular level, the infection strategies employed by L. boulardi and L. heterotoma appear to differ in distinct ways.

L. heterotoma actively suppresses host encapsulation of its eggs using VLPs produced within its venom glands (referred to as long glands). These VLPs enter the larval hemolymph along with the egg and quickly bind to host lamellocytes, become internalized, and promote morphological changes in the lamellocytes causing them to lyse [39]. Infection by L. heterotoma also results in apoptosis of pro-hemocytes in the lymph gland, and possibly of the circulating plasmatocytes as well [40]. *L. boulardi* also produce VLPs, but their morphology differs from those of L. heterotoma and they do not cause host lamellocyte lysis. Instead, L. boulardi venom (at least from common, virulent strains) appears to partially block the induction and release of lamellocytes from the lymph gland [41,42], and alters the morphology of a portion of the host's circulating lamellocytes (though not to the degree of L. heterotoma venom) [42–44]. Furthermore, while L. heterotoma eggs are found floating freely in host hemolymph, L. boulardi eggs are typically attached to host tissues, which provides a passive, physical protection against complete encapsulation by host hemocytes [45].

In this study, we document differences in the host ranges and cellular level infection characteristics of new L. heterotoma and L. boulardi strains from California (Lh14 and Lb17, respectively) that are highly virulent on wild-type D. melanogaster. To determine whether the VLP-containing venom of these parasites actively suppresses NF-κB signaling and other immune pathways in *Drosophila*, we used whole-genome microarrays to assess the transcriptional response of D. melanogaster larvae to attack by each wasp. We find, surprisingly, that D. melanogaster mounts a robust (albeit ultimately futile) immune transcriptional response against the specialist L. boulardi, similar in many respects to its successful immune transcriptional response against A. tabida, but that initiation of the immune transcriptional response is completely suppressed by the generalist L. heterotoma. We show that *Drosophila*'s immune response to L. boulardi infection incorporates both the cellular and humoral arms of the immune system, and the Toll pathway plays a central role in this coordinated response. Our study provides insight into the coordination of immune responses in the fly and provides clues to the mechanisms and evolution of parasite infection strategies.

## Results

### Lb17 and Lh14 Compared to Other Characterized Strains

**Figure 1:**
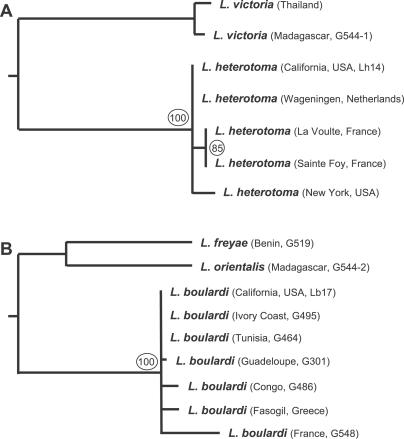
Phylogenetic Comparison of Lb17 and Lh14 to Other Characterized Strains Parsimony trees with bootstrap scores (circled) made from ribosomal RNA *ITS2* sequences show the close relationship between (A) Lb17 and (B) Lh14 with other strains of these species [37].

**Figure 2:**
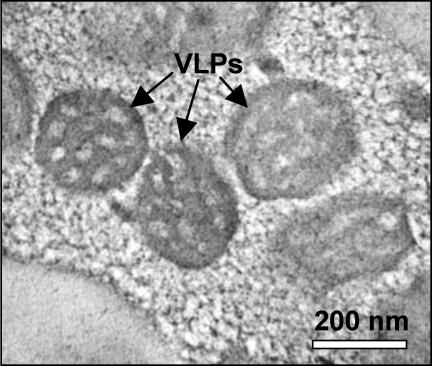
Lb17 VLPs TEM micrograph of the rounded, vesicle-filled VLPs from the lumen of the long gland reservoir of Lb17 shows that Lb17 VLPs are similar to those from other virulent L. boulardi strains [46].

Several studies have described the organismal and cellular level infection dynamics of L. heterotoma and L. boulardi in D. melanogaster hosts, mainly using wasp strains collected in Europe and Africa. To begin to characterize these interactions at the molecular level, we first generated new, inbred strains of L. heterotoma (Lh14) and L. boulardi (Lb17) collected from California, United States of America. DNA sequence from the ribosomal RNA *internal transcribed spacer 2* (*ITS2*) locus of Lh14 was similar to that from all other L. heterotoma strains, and was identical to that from a strain from Wageningen, Netherlands (Figure 1A) [36]. Furthermore, sequence data from Lb17 was similar to that from all other L. boulardi strains, and was identical to that from the virulent L. boulardi strains G464 from Tunisia and G495 from Ivory Coast (Figure 1B) [37]. One characterized L. boulardi strain, G486 from Congo, is distinct from other described strains in that it is relatively avirulent in immune-competent D. melanogaster larvae, and compared to virulent strains has relatively elongated VLPs with few vesicles [46] and a unique venom protein profile [47]. We found the ultrastructure of VLPs from the long gland of Lb17 to be very similar to the rounded, vesicle-filled VLP structure described from virulent strains of L. boulardi (Figure 2) [46], suggesting that Lb17 would also be very virulent in D. melanogaster.

### Host Range of Lb17 and Lh14

**Figure 3:**
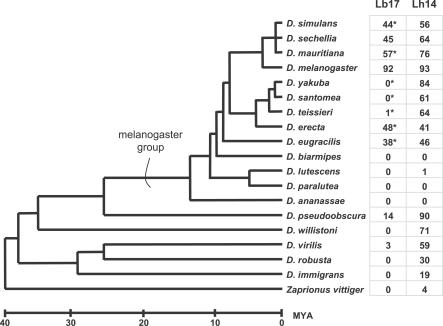
Infectivity of Lb17 and Lh14 on Multiple *Drosophila* Species Percentage of fly larvae that were successfully parasitized by the wasps (i.e., the percentage of fly larvae from which a wasp eventually hatched). Asterisks indicate that a significant proportion (>5%) of the fly larvae melanotically encapsulated wasp eggs or larvae. The *Drosophila* phylogeny is a consensus of multiple studies [73,113]; branch lengths are approximate.

In a review of disparate laboratory and field studies, L. boulardi was found to be especially infectious on D. melanogaster and its close relatives, while L. heterotoma was found to successfully infect a number of species across the *Drosophila* genus, causing them to be labeled specialist and generalist parasitoids, respectively [2]. In our study, we tested the ability of Lh14 and Lb17 to infect a wide diversity of *Drosophila* species under identical conditions. Although laboratory tests can only indirectly inform us of wasp host range in nature, we found Lb17 to be much more specialized than Lh14 (Figure 3).

**Figure 4:**
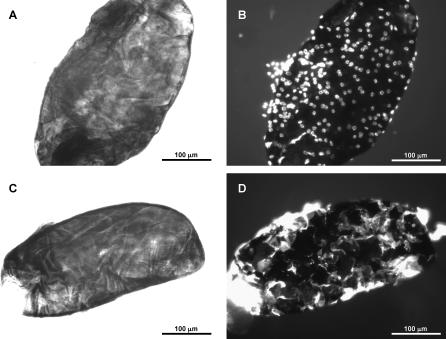
Melanotic Encapsulation of Lb17 Eggs by D. yakuba (A, C) White light pictures of melanized wasp eggs dissected from D. yakuba larvae. (B, D) Fluorescence pictures of the same melanized wasp eggs. (B) Hoechst nuclear stain—note abundance of cells encapsulating melanized egg. (D) Rhodamine-phalloidin actin cytoskeleton stain—note that encapsulating cells have flattened (lamellocyte) morphology.

Lb17 is adept at parasitizing the cosmopolitan fly species D. melanogaster (success rate >90%), and with moderate success can develop from certain other closely related species from the *melanogaster* group (∼50% success rate). However, Lb17 rarely successfully parasitizes flies from other Drosophila groups even under ideal conditions, and a significant fraction (>5%) of larvae from many *melanogaster* group species appear to melanotically encapsulate Lb17 eggs. Indeed, staining of melanized wasp eggs from one such species, D. yakuba, showed there was an abundance of lamellocytes surrounding the melanized egg (Figure 4). Thus, at least in some host species, Lb17 infection activates the stereotypic cellular immune response characterized by changes in hemocyte activation, migration, adhesion, and melanization. On the other hand, Lh14 successfully parasitizes all of the species that Lb17 can infect, is almost never melanotically encapsulated, and can also successfully infect a wide range of *Drosophila* species and groups immune to Lb17 infection. Thus, Lh14 is a generalist of the genus *Drosophila*, while Lb17 appears to specialize on D. melanogaster and its close relatives. For simplification, we will hereafter use the relative terms “generalist” and specialist” for these two wasp species, though L. heterotoma is less of a generalist and L. boulardi less of a specialist than many other parasitoid wasp species.

### Organismal and Cellular Level Infection Characteristics Differ between Lb17 and Lh14

**Figure 5:**
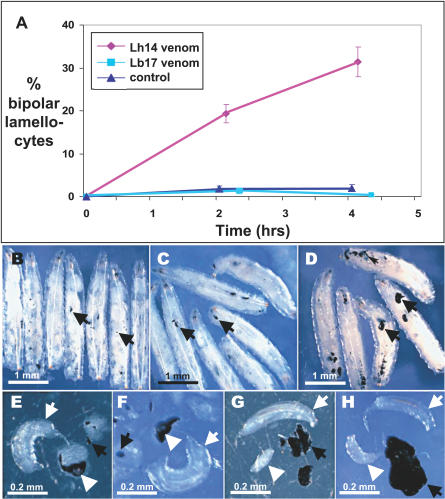
Different Phenotypic Effects of Lb17 and Lh14 Venom (A) Percentage of *Drosophila* lamellocytes lysed (bipolar) after incubation with wasp venom in vitro (means and standard errors shown). (B) *hop^Tum-l^* larvae form melanotic tumors (black arrows), even when infected with either Lb17 (C, E, F) or Lh14 (D, G, H). Though dominant larvae of neither wasp species are encapsulated (white arrows), melanization of supernumerary wasp larvae (white arrowheads) occurs only in Lb17 attacked *Drosophila*.

To gain insight into the mechanisms responsible for the difference between Lb17 and Lh14 in ability to survive in Drosophilia hosts, we examined their infection characteristics in D. melanogaster in detail. First, we incubated lamellocytes from D. melanogaster tumor strain (*hop^Tum-l^*) larvae in vitro with fluid derived from the long gland of each wasp species. We found a striking difference in the percentage of lamellocytes undergoing lysis. As expected, Lh14 venom severely compromised lamellocyte morphology and viability, but Lb17 venom had no observable effect on lamellocyte morphology in this time frame (Figure 5A). We next allowed wasps to lay supernumerary eggs in single *hop^Tum-l^* larvae, and found that dominant wasp larvae of neither species were encapsulated in the *hop^Tum-l^* hosts. However, unlike Lb17, dead supernumerary Lh14 larvae were also protected from melanotic encapsulation (Figure 5B–5H). Finally, Lh14 eggs are usually found floating freely in host hemolymph, while Lb17 eggs are invariably attached to host tissues (gut, fat body), suggesting that Lb17 passively insulates itself from complete encapsulation [45].

These results demonstrate that Lh14 (like other L. heterotoma strains) utilizes a highly immune suppressive infection strategy characterized by destruction of host hemocytes, while Lb17 utilizes a different strategy that appears to combine passive immune evasion with a relatively weak or localized suppression of melanotic capsule formation.

### Overall Host Transcriptional Response to Lb17 and Lh14 Infection

To test the downstream effects of these differences in wasp infection strategies, as well as to reveal the molecular basis of *Drosophila*'s response to parasitoid wasps, we undertook a genome-wide gene expression study of D. melanogaster larvae attacked by Lb17 and Lh14 at three time points post-infection (2–5 h, 9–12 h, and 21–24 h). These time points correspond to important events in the successful encapsulation of a parasitoid egg: hemocyte activation and hematopoiesis, hemocyte differentiation into lamellocytes, and wasp egg encapsulation and melanization, respectively [8,42,48]. We tabulated the numbers of genes showing significant expression level differences for four treatment comparisons: Lb17 attacked flies versus control, Lh14 attacked flies versus control, merged Lb17 and Lh14 attack datasets versus control, and Lb17 attack versus Lh14 attack. We also estimated the false positive rate (*q*-value) for the set of genes identified under each *p*-value cutoff (Table 1).

**Table 1:**
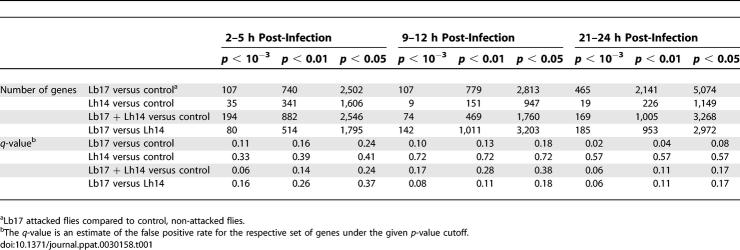
Number of Differentially Regulated *Drosophila* Genes after Wasp Attack for Given *t*-Test *p*-Value Cutoffs

We first focus on gene expression differences in the Lb17 attack versus control and Lh14 attack versus control treatment comparisons (Table 1). Despite nearly identical infectivities of Lb17 and Lh14 in this experiment (1.27 and 1.29 wasp eggs per larva, respectively; Materials and Methods), it is immediately clear that far smaller numbers of *Drosophila* genes are significantly differentially regulated by Lh14 attack than by Lb17 attack, and this discrepancy intensifies at later time points post-infection. For example, at a *p*-value cutoff of *p* < 10^−3^, 107 genes were up- or down-regulated in flies attacked by Lb17 5 h post-infection compared to only 35 for Lh14 attacked flies. At 12 h post-infection the ratio is 107 to 9, and at 24 h post-infection the ratio is 465 to 19. To further illustrate this point, we calculated the number of cases in which genes were 2-fold up- or down-regulated by Lb17 or Lh14 relative to control at all time points, and that were also significant at the *p* < 0.01 level. 97% of 471 such occurrences (involving 405 genes) were caused by Lb17 attack, mostly due to gene up-regulation (unpublished data). Clearly, D. melanogaster has a much more robust transcriptional response to attack by the specialist Lb17 than to the generalist Lh14, even though eggs of neither wasp are encapsulated by these hosts.

### Sorting Over- and Underexpressed Genes by Biological Function

To understand the biological effects of these transcriptional differences between Lb17 and Lh14 attacked flies Table 1), we first used a liberal criterion for identifying significantly differentially regulated genes: individual gene expression differences between treatments were considered significant if nominal (i.e*.*, not corrected for multiple tests) p < 0.01. We then used gene ontology (GO) classifications to determine whether our significantly differentially regulated genes were overly clustered in particular biological function categories, such as “defense response”. Specifically, we analyzed which *Drosophila* biological function categories were significantly overrepresented by genes showing significant expression level differences between specialist attacked and generalist attacked flies (Lb17 versus Lh14), and which biological function categories were overrepresented after attack by both wasp species (merged Lb17 and Lh14 datasets versus control). The latter comparison is more sensitive than separately analyzing Lb17 and Lh14 datasets alone versus control, due to the increase in power gained by increasing the number of replicates (Tables 1, S1, and S2).

We first identify the biological function categories that were overrepresented by differentially expressed genes from the treatment comparison Lb17 + Lh14 versus control. The *Drosophila* biological function categories most significantly up-regulated by both wasp species are proteolysis and energy generation, while the biological function category most significantly down-regulated by both wasp species is development (see Table S1 for numbers of genes from each category, and Table S2A for gene identities). Thus, one common response to wasp parasitism involves activation of proteolytic cascades, which are widely believed to be important for extracellular signaling in general, and for hemolymph coagulation and humoral immune signaling in particular. As very few such immune response proteolytic enzymes have been functionally studied in *Drosophila* [49–51], this list provides a set of candidates for future research. The second common response to parasitoid attack appears to involve conserving energy by slowing down normal cellular and physiological activities, and to instead devote molecular machinery to elevated ATP production via overexpression of genes involved in mitochondrial electron transport and oxidative phosphorylation. This energy production may help pay the cost of mounting an immune response. Expression of genes involved in the development of adult morphological structures such as eyes and gonads are especially down-regulated. This developmental slowdown is obvious at the organismal level, as infected D. melanogaster pupate approximately 2 d later than controls.

Only two major biological function categories, proteolysis (a novel set distinct from those discussed above) and defense response, show significantly different expression levels in Lb17 versus Lh14 attacked *Drosophila* at the early time points post-infection (2–5 h, 9–12 h), almost entirely due to gene up-regulation in Lb17 attacked flies (Table S2b). To a lesser extent, mRNA levels from two biological function categories important for wound healing, chitin metabolism and cuticle structural constituents, decreased initially but then were significantly up-regulated specifically in Lb17 attacked flies at the later time points (9–12 h, 21–24 h). Thus, at the molecular level, the main difference between Drosophila attacked by Lb17 and Lh14 is that Lb17 attacked flies appear to mount sustained immune and wound responses, within 12 and 24 h, respectively, while Lh14 attacked flies do not.

### Candidate Anti-Parasite Immune Response Pathways and Genes

**Figure 6:**
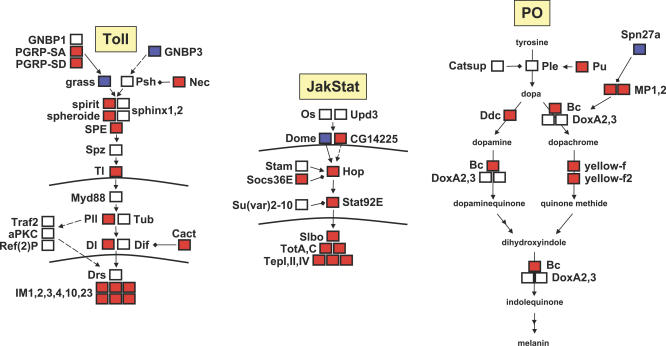
Regulatory Changes in Canonical Immune Pathways after Lb17 Attack Genes in red are significantly overexpressed and in blue underexpressed by Lb17 attacked flies in at least one of three treatment comparisons (Lb17 versus control, Lb17 + Lh14 versus control, or Lb17 versus Lh14) at one of the two early time points post infection (2–5 or 9–12 h). Dashed arrows represent presumed pathway interactions. Horizontal lines represent cell and nuclear membranes; genes inside the nucleus are targets of the upstream pathway. The main Toll pathway AMP Drosomycin was more than 4-fold up-regulated after Lb17 attack, but this change was not significant due to high variance among control replicates. Of the 33 overexpressed genes represented in this figure, only two, *PGRP-SA* and *PGRP-SD*, were also overexpressed in the Lh14 versus control comparison (relative to 21 in the Lb17 versus control comparison).

Several genetic pathways and individual genes have been implicated in the cellular arm of the *Drosophila* immune response through their involvement in hematopoiesis, hemocyte differentiation, hemocyte cytoskeletal rearrangements, and melanization (Introduction). We focus first on transcriptional differences in these known cellular immunity genes after Lb17 attack (Table S3). We found that numerous genes from the genetically linked Toll, JAK/STAT and PO pathways [50,52,53] are up-regulated in Lb17 attacked flies, confirming that the Toll pathway plays a central role in the anti-parasite immune response (Figure 6) [22]. The *Pvr* (VEGF receptor) ligands *Pvf1* and *Pvf2*, which are involved in hematopoiesis and hemocyte migration [26,54], were also significantly up-regulated within 12 h after infection by Lb17-attacked flies. *He*, a negative regulator of the cellular encapsulation response, was significantly up-regulated after attack by Lb17 at the later time points post-infection, possibly as part of the normal tamping down of hematopoiesis after an immune response is mounted. Neither Lb17 nor Lh14 attack induced obvious transcriptional changes in the Imd, JNK, or Wnt genetic pathways (Table S3), or in other candidate encapsulation response genes. In fact, the Imd pathway members *Dredd* and *Relish* were both significantly down-regulated within 12 h of Lb17 infection.

Although not yet cloned, *Drosophila* loci important for variation in resistance to two different wasp species, L. boulardi and A. tabida, have been classically mapped to cytological positions 55C-F and 35B-E and are named *Rst(2)Lb* and *Rst(2)At*, respectively (the cytological position of *Rst(2)At* was inferred via conversion from genetic position 2.51–3, http://www.flybase.org/) [55]. Interestingly, expression of genes known to be regulated by the Toll and JAK/STAT pathways occur in both of these regions, and are some of the most highly expressed genes after Lb17 attack identified in our survey (Table S3). The 55C-F region contains a large cassette of tightly linked and coregulated “immune induced” genes thought to be regulated by the Toll pathway [5], several of which are greatly up-regulated after Lb17 attack but not Lh14 attack. Likewise, the gene *edl*, which was previously implicated in the immune response against L. boulardi [56], plays a regulatory role in and is itself regulated by the Ras pathway signaling [57], and was more than 2-fold up-regulated after Lb17 attack but not Lh14 attack. In the 35B-E region, the Toll/JAK/STAT-regulated complement-like protein *TepI* [52] is greater than 10-fold up-regulated after Lb17 attack, but is not differentially regulated after Lh14 attack. An important role for *TepI* in the *Drosophila* antiparasite immune response is further supported by the fact that a mosquito Tep protein binds to and mediates melanotic killing of malarial ookinetes [58].

One of the last stages in a successful encapsulation response is the migration and adhesion of new hemocytes (particularly lamellocytes) to the wasp egg, the flattening and spreading of hemocytes over the egg, and formation of septate junctions among hemocytes to tightly seal the melanotic capsule [8]. It is at this stage that the *Drosophila* immune response to L. boulardi appears to break down, as lamellocytes lose their flattened shape and fail to adhere to the wasp egg or to each other [41,42]. Interestingly, there is a small but significant excess of genes involved in both cell migration and cell adhesion down-regulated in response to both wasp species at the 24-h time point, when encapsulation is thought to occur. Thus, it is possible that Lb17 and Lh14 actively suppress encapsulation by regulating *Drosophila* lamellocyte membrane components and cytoskeletal structure, strategies previously observed in other parasitoid systems [59].

Cell migration requires three steps: the generation of an actin-rich lamella at the leading edge of the cell, adhesion of the advancing leading edge to the extracellular substrate, and release from the extracellular substrate by the trailing edge of the migrating cell [60,61]. A recent study identified 21 proteins necessary for proper lamella formation, the first step in cell migration, in *Drosophila* S2 (immune-competent) cells [62]. Remarkably, nine of these 21 genes were significantly down-regulated in Lb17 attacked flies at the 21–24-h time point post-infection, four of which are thought to function in actin nucleation and branching as part of the Arp2/3 and SCAR complex (*Abi*, *Arp2*, *kette*, *Sra-1*). None of these 21 genes were significantly down-regulated in Lh14 attacked flies (Table S3), suggesting that inhibition of lamella formation may be unique to Lb17 infection. Several of the cell migration genes down-regulated after attack by both wasp species are thought to play roles in adhesion of migrating axons to the extracellular substrate and may play similar roles during hemocyte migration. These include an overlapping group of genes with immunoglobulin (*Dscam*, *Lar*, *leak*), fibronectin (*dome*, *Dscam*, *Lar*, *leak*, *Ptp99a*), and protein tyrosine phosphatase (*csw*, *dome*, *Lar*, *Ptp99a*) domains, along with the interacting protein tyrosine kinase gene *Abl* (a homolog of which is involved in T cell–mediated immunity in mice [63]). In particular, *Dscam* encodes a massively alternatively spliced protein recently found to be expressed in hemocytes as well as in secreted form in the hemolymph, and RNA interference of *Dscam* mRNA results in decreased ability of hemocytes to phagocytose bacteria [64].

Of the down-regulated cell adhesion genes, two are well-characterized homophilic cell adhesion genes important for motor axon fasciculation (*Con*, *Fas2*) [65]. Several genes important for septate junction assembly were also specifically down-regulated in Lb17 attacked flies (*Cont*, *cora*, *crb*, *dlg1*, *Lac*, *Nrg*, *Nrx-IV*, *pck*, *sinu*). Whether any of these cell migration and cell adhesion genes function in hemocyte migration and attachment during the cellular immune response is not yet known, but the fact that many of them were originally identified from neurons is interesting given that notable parallels between nervous system development and the immune response have recently come to light [64,66].

### Experimental Confirmation of Microarray Results

**Figure 7:**
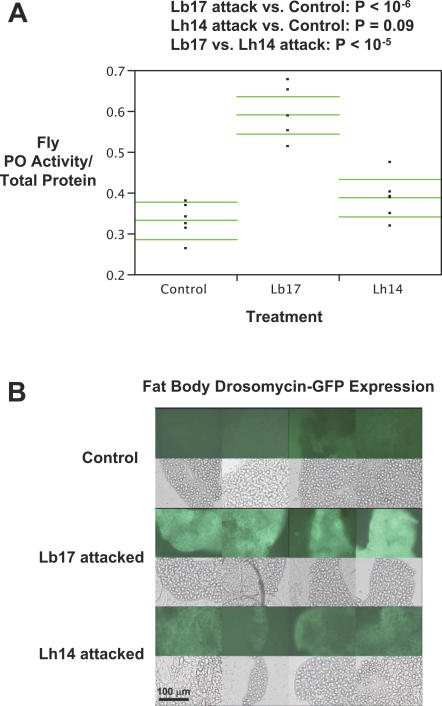
Confirmation of Microarray Results at the Protein Level (A) PO enzyme activity (standardized by total protein levels) in *Drosophila* larvae 24 h after wasp attack. Means and upper/lower 95% confidence intervals are represented by horizontal lines. (B) Fat body expression of Drosomycin-GFP 24 h after wasp attack.

To confirm our microarray results showing distinct differences in the *Drosophila* immune response against Lb17 and Lh14, we tested whether PO activity and NF-κB pathway activation were induced in Lb17 attacked flies in independent experiments. First, we assayed host hemolymph PO enzyme activity after wasp attack by in vitro measurement of the conversion of L-DOPA to dopachrome (Materials and Methods). We found significantly increased PO activity in hemolymph form Lb17 attacked larvae 24 h post-infection, while PO enzyme activity in Lh14 attacked *Drosophila* larvae could not be distinguished from control (Figure 7A). These data mimic our microarray results at the protein level, i.e., the increased transcription of the PO pathway genes *Pu*, *Ddc*, *Bc*, *yellow-f*, *yellow-f2*, and prophenoloxidase (proPO) activating enzymes (and decreased transcription of a negative regulator of the PO pathway, *Spn27a*) at the earlier time points is associated with increased PO enzyme activity in Lb17 attacked fly hemolymph 24 h post-infection, when melanotic encapsulation typically occurs [8].

Next, we assayed host fat body production of green fluorescent protein GFP under the control of several antimicrobial peptide (AMP) promoters after attack by both wasps. We found that a significantly greater proportion of fat body cells express the Toll pathway–specific Drosomycin-GFP in Lb17 versus Lh14 attacked larvae (Table 2), and that even in GFP-producing cells GFP fluorescence was markedly higher in Lb17 attacked flies (Figure 6B). We also found significant production of the partially Toll pathway–regulated Metchnikowin-GFP after attack by both wasp species [67], and no detectable production of Cecropin A1-GFP, Diptericin-GFP, or Drosocin-GFP after attack by either wasp species, results that are all consistent with the microarray gene expression results (Figure S1). Attacin-A gene expression was highly up-regulated by both wasp species 5 h post-infection before returning to normal levels at later time points, but we did not find an increase in fat body production of Attacin-A-GFP protein at the same early time point (Figure S1). The Drosomycin and Metchnikowin GFP results show that the *Drosophila* transcriptional response to wasp parasitism is not limited to the cellular response immune system tissues, but incorporates elements of the fat body–derived humoral response as well.

**Table 2:**
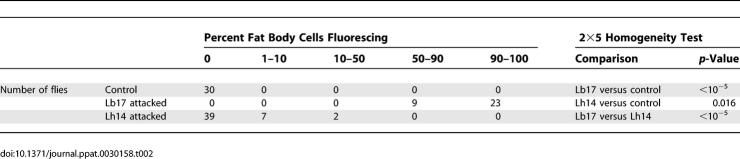
Percent of Fat Body Cells Fluorescing in the Drosomycin-GFP Strain 5 h after Wasp Attack

## Discussion

### Intraspecific Variation in Wasp Infection Strategies?

Although it is rare to find L. boulardi and L. heterotoma strains in the wild that are not completely virulent on D. melanogaster [38,68], some exceptions have been documented [69,70]. In particular, L. boulardi strain G486 (derived from a collection from Congo in 1977) and strains from other tropical African populations are sometimes encapsulated by D. melanogaster [69,71]. This may be due to fitness tradeoffs associated with wasp specialization on other *melanogaster* subgroup host species present in Africa, such as D. yakuba [72]. Other presumably correlated phenotypic differences between G486 (and its derivatives) and virulent L. boulardi strains are (a) VLPs of the avirulent strain hold fewer vesicles and are more oblong [46], (b) natural infection by the avirulent strain does not result in alteration of host lamellocyte morphology, while infection by virulent strains results in approximately one-third of host lamellocytes having distorted shapes [42,45], and (c) infection by virulent strains results in somewhat lower lamellocyte induction compared to the avirulent strain [41,42]. These differences suggest it is possible our characterizations of the wasp infection strategies are specific to the particular wasp strains we used.

However, for reasons described below, we assume that the infection mechanisms employed by L. boulardi and L. heterotoma are relatively consistent within each species, that any variation within the wasp species is minimal compared to the differences between species, and that data from Lb17 and Lh14 are representative of L. boulardi and L. heterotoma, respectively. G486 is only encapsulated at significant frequency by D. melanogaster strains artificially selected for increased encapsulation ability [69], and is quite virulent on wild-type D. melanogaster strains such as Oregon R, suggesting that in natural conditions there is little variability in L. boulardi success in D. melanogaster hosts. Furthermore, the differences between G486 and the “virulent” L. boulardi strains may be caused by genetic variation in a single VLP component [47] responsible for the species specificity of the infection strategy [72], and may not represent wholesale changes in the infection strategy itself (i.e., the mechanism by which the primary host's immune system is suppressed and/or evaded). G486 and virulent L. boulardi strains also share many attributes that distinguish them from L. heterotoma: (a) both virulent and avirulent L. boulardi strains lay eggs attached to host tissues [45] (Results), (b) natural infection of D. melanogaster larvae using both virulent and avirulent L. boulardi strains results in an increased abundance of circulating plasmatocytes and lamellocytes [42,43], and (c) dead supernumerary larvae of both virulent and avirulent L. boulardi strains are melanotically encapsulated in tumorous fly strains [45] (Results). Finally, we have shown that Lh14 and Lb17 are closely related to other characterized virulent wasp strains (Figure 1) and that Lb17 VLPs are morphologically identical to VLPs from other virulent L. boulardi strains (Figure 2), suggesting our results using the Lb17 and Lh14 strains are indicative of the typical interactions between these wasp species and their *Drosophila* hosts.

### Differences in Infection Characteristics between L. boulardi and L. heterotoma

While successful infection by L. boulardi is mainly limited to hosts from the *melanogaster* group, L. heterotoma successfully infects flies of diverse sizes and developmental times across the entire genus *Drosophila*, which may be as old as 40 million years [73]. This broad host range is particularly impressive for an endoparasite, which must synchronize its own development and nutritional requirements with that of its host [74,75]. Virulent strains of both L. boulardi and L. heterotoma use immune suppression to overcome the immune response of their hosts, as evidenced by the protection their venom gives to secondary parasites in wild-type *Drosophila* larvae [2,44,76–78]. However, this immune suppression is achieved in very different ways. We make the assumption that differences in the effects of L. boulardi and L. heterotoma venom in D. melanogaster hosts can help us understand why these wasps have such varying levels of success in other host species.

D. melanogaster initiates an immune response against both L. boulardi and L. heterotoma by undergoing a rapid burst of hematopoiesis [42,43,79]. However, L. heterotoma venom causes rapid lysis of the circulating host lamellocytes and prevents further release of lamellocytes by apoptosing hemocyte precursors in the lymph gland [40]. In combination, these effects lead to a near complete lack of circulating lamellocytes in L. heterotoma attacked wild-type flies [70,80]. Furthermore, our microarray results demonstrate that infection by L. heterotoma results in a near complete failure of attacked flies to mount an immune transcriptional response, as if these flies do not recognize that they are infected. The extremely immune suppressive effects of L. heterotoma venom prevent highly immune-competent (tumorous) D. melanogaster hosts from melanotically encapsulating dead supernumerary wasp larvae (Figure 5), and may be largely responsible for the broad host range of this parasite species.

L. boulardi relies instead, at least partially, on a passive immune evasion strategy of attaching its eggs to host tissues. Its venom can limit lamellocyte induction [41] and cause moderate alterations in host lamellocyte cytoskeletal structure such that the lamellocytes fail to adhere to the wasp egg, but host lamellocytes are not lysed [45]. This suppression of melanotic capsule formation by L. boulardi is limited to live L. boulardi tissues (at least in highly immune-competent tumorous hosts), suggesting that some important immune-suppressive venom components are limited to the wasp eggs/larvae themselves (e.g., [81]). Furthermore, L. boulardi clearly differs from L. heterotoma in that the numbers of host plasmatocytes and lamellocytes both significantly increase in wild-type flies after L. boulardi attack [42]. As we show here, this increase is accompanied by a robust host immune transcriptional response incorporating both the cellular and humoral arms of the immune system and several characterized immune pathways. Thus, L. boulardi venom is far less immune suppressive than that of L. heterotoma. The limited host range of L. boulardi relative to L. heterotoma might be explained if L. boulardi's relatively benign alteration of *Drosophila* lamellocyte morphology requires a higher degree of venom specificity than hemocyte destruction.

If host hemocyte lysis is a major cause of immune system stasis in L. heterotoma attacked flies, circulating hemocytes would seem to play an important role in initiating and coordinating *Drosophila*'s integrated immune response, presumably through the release of proteolytic enzymes and other extracellular effector proteins [82,83]. Interestingly, dispersed hemocytes are especially numerous in the posterior region of the dorsal vessel in unchallenged *Drosophila* larvae [48], in the same region L. boulardi and L. heterotoma almost invariably inject their eggs. However, the near-complete immune system stasis of L. heterotoma attacked flies suggests that this parasite may utilize a cocktail of venom components and mechanisms for inhibiting host immune responses. It is already clear that structural lysis of host lamellocytes and apoptotic killing of host hemocyte precursors in the lymph gland occur via different mechanisms [40]. So, it would not be surprising to find that L. heterotoma venom could also specifically inhibit Toll or Imd pathway signaling in the fat body, block PO activation, or have other specific deleterious effects in infected hosts. Indeed, in other host–parasitoid systems, wasp-injected polydnaviruses encode proteins known to inhibit NF-κB signaling [84] and to limit PO enzyme titers [85].

Although the D. melanogaster immune transcriptional response is essentially silenced by L. heterotoma infection, other aspects of *Drosophila*'s regulatory response to infection are shared in *L. boulardi–* and L. heterotoma–infected flies. For example, development-related genes are down-regulated after attack by both wasps, whereas genes involved in ATP generation are up-regulated after attack by both wasps. Whether the reallocation of ATP resources after wasp infection benefits the host by paying the cost of mounting an immune response, benefits the parasitoid by providing resources for rapid development, or is simply a cost of the trauma of parasitism that benefits neither party, these data provide molecular corroboration for the substantial energetic fitness costs associated with parasite infection [35,86,87], and help establish *Drosophila* as a model for immune-related energy metabolism (e.g*.*, [88,89]). Furthermore, the substantial concordance in *Drosophila* gene regulation after attack by each wasp (outside of immune response genes) shows that the relative paucity of differentially regulated genes in Lh14 attacked flies cannot entirely be due to a general inhibition of host transcription, but is at least partly due to a specific inhibition of immune response induction.

### The D. melanogaster Immune Response against L. boulardi Infection

During the course of this study, another group documented the transcriptional response of *Drosophila* to attack by a relatively avirulent strain of a Braconid parasitoid wasp, A. tabida, which D. melanogaster successfully encapsulates 73% of the time [32]. Like L. boulardi, A. tabida uses an at least partially immune evasive infection strategy manifested by “sticky” eggs that become embedded in host tissue, but unlike L. heterotoma and L. boulardi, A. tabida venom does not contain VLPs [33]. Wertheim et al. took a conservative approach to assessing significance of potentially differentially regulated genes, and thus reported only 159 *Drosophila* genes differentially expressed after A. tabida attack [32]. Expression changes in these 159 genes after attack by Lh14 and Lb17 are listed in Table S3. When we compared *Drosophila*'s (usually unsuccessful) transcriptional response against Lb17 to its (usually successful) transcriptional response to A. tabida [32], we found that a significant proportion of the 159 differentially regulated genes identified by Wertheim et al. were also differentially regulated in our study, especially for genes within the defense response functional category (up to 74%, Tables S3 and S4). Thus, the L. boulardi infection strategy appears to leave much of the host immune system intact, and is focused instead on blocking or evading only the very last steps of the *Drosophila* immune response.

D. melanogaster up-regulates a surprisingly high proportion of canonical Toll, JAK/STAT, and PO pathway genes within 12 h after infection by Lb17 (Figure 5; Table S3). Our findings are consistent with previous work showing that mutations in genes from the Toll and JAK/STAT pathways have strong effects on hemocyte concentration, lamellocyte differentiation, and wasp egg encapsulation [22], and that mutations in PO cascade genes result in the inability to melanize encapsulated wasp eggs [18]. Because (a) Toll signaling controls PO pathway activity [50,53], (b) the Toll pathway is involved in crosstalk with the JAK/STAT pathway [52,53], (c) functional genetic studies using Toll pathway mutants have shown that the Toll pathway is required for the melanotic encapsulation based immune response [22], and (d) because up-regulation of components from these pathways is the main difference between L. boulardi and L. heterotoma attacked flies, the Toll pathway appears to be a central regulator of the anti-parasite innate immune response.

Although all three of the major immune tissues (lymph gland, hemocytes, fat body) in *Drosophila* larvae are clearly involved in the response to wasp infection, many questions remain regarding the specific roles played by each tissue, and whether the Toll, JAK/STAT, and PO pathways have important functions in each. Using transgenic flies, we showed that the Toll-regulated *Drosomcyin* and *Metchnikowin* promoters are activated in the D. melanogaster fat body (a humoral response immune tissue) after infection by L. boulardi, whereas the Diptericin promoter and other Imd pathway–regulated AMP promoters are not. Post-infection transcription levels of *Drosomycin* decline over time, which may explain why no change in Drosomycin expression levels was found in L. boulardi attacked flies in a previous study using longer post-infection time points [90]. Thus, our data show that *Drosophila*'s transcriptional reaction to wasp parasitism incorporates elements of both the cellular and humoral responses. Although fat body antimicrobial peptide production may only be a generic defense response to the cuticle wound caused by the wasp ovipositor, or a byproduct of humoral activation of the Toll pathway for other reasons, and not an immune response to the parasite itself [5,91], it is striking that Lh14 infection appears to result in the specific suppression of fat body activation of the Drosomycin promoter, a well-characterized downstream effector of the Toll pathway [92]. This result raises the possibility that immune peptide secretion by the fat body is dependent on the hemocytes inactivated by Lh14 venom, or that Lh14 venom specifically interferes with Toll pathway signaling in the fat body.

Analysis of the microarray data indicated that expression of several components of the melanization pathway increased after Lb17 infection (Figure 5; Table S3), in concordance with a similar result from flies infected by A. tabida [32]. Consistent with our gene expression data, we showed that infection by Lb17 results in significantly elevated PO enzyme activity in D. melanogaster hemolymph. Taken together, our gene expression level data and enzymatic assays clearly demonstrate that hemolymph PO enzyme activity is elevated in Lb17 attacked flies post-infection, and that Lh14 is able to suppress hemolymph PO activation. These results seem to contradict a previous report that L. boulardi venom components “impair the melanisation pathway” in *hop^Tum-l^* fly larvae [44]. In that study, both the proportion of flies showing tumors and the strength of melanization of existing tumors declined after attack by L. boulardi [44]. Two potential explanations for this apparent discrepancy are that (a) Lb17 venom may block conversion of proPO into PO, which we cannot distinguish in our assay (Materials and Methods), or (b) hemocytes, which are thought to control release of PO enzymes [93], may be recruited away from otherwise tumorous *hop^Tum-l^* self tissue toward L. boulardi eggs after infection. It is also interesting to note the apparent increase in size of melanotic tumors in larvae attacked by Lh14 (Figure 3D), which may be an indication that L. heterotoma venom induces lysis of PO-bearing hemocytes clumped at the site of a tumor.

In contrast to the Toll, JAK/STAT, and PO pathways, we were surprised to find that regulation of Imd, JNK, and other described *Drosophila* immune pathways appears largely unaffected by wasp infection, at least at the time points post-infection that we assayed (Table S3). These findings are consistent with the demonstration that *Relish* null mutant flies retain the ability to melanotically encapsulate and kill parasitoid wasp eggs [94], but are potentially at odds with the demonstration that Bsk (a member of the JNK pathway) is required for lamellocyte activation and the encapsulation response [28]. Thus, different scenarios may explain the lack of gene expression changes in these pathways: (a) certain pathways may play no role in the anti-parasite immune response, (b) signaling through certain pathways does occur after wasp attack but this contribution is not reflected in gene expression changes, or (c) certain pathways normally play a role but their up-regulation is specifically suppressed by Lb17 and Lh14 venom. With the exception of *Relish*, which was also slightly up-regulated at the early time point our study, none of the Imd or JNK pathway genes was significantly up-regulated in *D. melanogaster* after attack by a relatively avirulent A. tabida strain either [32], casting doubt on the latter hypothesis.

It is still unclear what induces the *Drosophila* anti-parasite immune response, whether it is the cuticle wound caused by the wasp ovipositor, the wasp egg itself, components of the venom, or a combination of these [48,91]. It seems likely that that the injury caused by the wasp ovipositor is enough to trigger at least the initial burst of hematopoiesis in attacked flies [41,91]. The recruitment of *Drosophila* hemocytes toward the wasp egg shortly after infection also makes clear that the wasp egg is quickly recognized as foreign, perhaps as a result of being flagged by extracellular recognition proteins. Our microarray results point to at least two strong candidate pattern recognition receptors for wasp eggs, *TepI* and *Lectin-24A*. *TepI* is located at cytological position 35D6, which was classically mapped as a chromosomal region harboring natural variation for resistance against the parasitoid wasp A. tabida [55]. Lectins have long been supposed to play a role in innate immunity pattern recognition because they can bind to very specific cell membrane sugar moieties [95]; however, no such lectins were found overexpressed in the *Drosophila* antimicrobial microarray studies [6,7]. Both *TepI* and *Lectin-24A* were more than 10-fold up-regulated in response to Lb17 infection, and were also significantly up-regulated in response to attack by A. tabida [32]. Though flies attacked by L. heterotoma initiate an immune response, they fail to up-regulate these genes, suggesting L. heterotoma attack results in suppression of host anti-parasite immune recognition capability.

We also identified a set of novel candidate *Drosophila* anti-parasite immune response genes involved in cell migration and cell adhesion. These genes were down-regulated after attack by both wasps at a time point post-infection (∼24 h) when wasp egg encapsulation by *Drosophila* hemocytes typically occurs. Although we do not know which host cells show the most significant infection-induced cytoskeletal changes (following these gene expression changes), the idea that Lb17 and Lh14 actively suppress encapsulation by regulating *Drosophila* lamellocyte cytoskeletal structure is consistent with the previous observations that (a) the effects of L. heterotoma venom on lamellocytes were found to be blocked by microtubule inhibitors [96], and (b) L. boulardi venom contains a Rho-GAP protein, which, when purified and injected into flies, resulted in significant alterations in lamellocyte cytoskeletal morphology [41,47]. However, if we assume that these gene expression changes are not limited to hemocytes (hemocytes represent very few cells in whole larvae), our data suggest that infection (and venom factors) elicit changes in other host organs and tissues, whose identity remains to be determined, e.g*.*, [46]. Notable differences in cell migration and cell adhesion gene regulation between Lb17 and Lh14 attacked flies were also found. For example, only Lb17 attacked flies showed significant down-regulation of genes involved in lamella architecture and septate junction formation. This finding is consistent with the hypothesis that L. boulardi relies on highly specific alterations in host lamellocyte cytoskeletal structure as opposed to killing host lamellocytes outright.

### Evolution of Parasitoid Wasp Infection Strategies

L. boulardi and L. heterotoma are closely related parasites with overlapping host ranges and overlapping geographic ranges, yet they overcome the immune response of at least one common host (D. melanogaster) in remarkably different ways. Given that activation of the host immune response likely depletes the energetic resources of the host, and thereby the developing parasite, the L. boulardi infection strategy appears to be disadvantageous. There are at least two potential explanations for the specialist's less immune-suppressive infection strategy. First, host wounding caused by wasp oviposition, as well as a prolonged developmental period, means that parasitized host larvae may be more susceptible to secondary pathogenic infections than unparasitized flies [97,98]. Thus, L. boulardi, which appears to heighten its host's standing immune defenses but is protected from the immune response itself, may be at a comparative advantage to a “clumsy” parasite that must disable its host's immune response in order to survive [45].

Second, highly immune suppressive venom may be costly to produce, incurring a fitness tradeoff to the generalist L. heterotoma. For example, L. heterotoma (but not L. boulardi) eggs succumb to melanotic encapsulation more frequently as females attack more hosts, suggesting that generalist females “run out” of venom [44,99,100]. Furthermore, the specialist L. boulardi is known to outcompete L. heterotoma for shared D. melanogaster hosts in laboratory experiments [76]. Since supernumerary wasp larvae are usually killed by the first wasp larva to hatch [2,101], high competitive success of L. boulardi can be explained by the fact that L. boulardi eggs hatch approximately 10 h earlier than L. heterotoma eggs (∼40 h versus ∼50 h post-infection at 23 °C, unpublished data). Such opportunity for competition between wasp larvae/eggs inside hosts in nature is high, as up to 40% of fly larvae collected in the field are multiply parasitized [3]. This critical difference in wasp developmental time leads to the hypothesis that there is an evolutionary tradeoff between devoting resources to highly immune suppressive venom (and increased parasitization success) and devoting resources to rapidly developing eggs (and increased competitive ability).

L. boulardi and L. heterotoma are distinguished by a suite of presumably coadapted infection characteristics. For example, L. heterotoma lays unattached eggs, destroys host hemocytes, blocks Toll pathway signaling in the fat body, is a slow developer, and has a broad host range. Whether such traits typically coevolve in generalist parasitoids, and whether it is a general pattern that specialist pathogens evolve to become less immune suppressive and more immune evasive over time remains an open question. Interestingly though, some of the differences between L. boulardi and L. heterotoma seem roughly paralleled in a pair of Braconid wasps, A. tabida and A. citri, which can also infect D. melanogaster. Unlike A. tabida, A. citri lays unattached eggs, disrupts host hematopoiesis and melanogenesis, is a slow developer, and is melanotically encapsulated by D. melanogaster and D. simulans at lower frequency than A. tabida (though no direct comparison of host range between A. tabida and A. citri has been made) [102–104]. A much larger number of independent generalist and specialist parasite lineage pairs will need to be identified and examined to determine whether specialist pathogens typically adopt less immune suppressive strategies. However, should such a pattern exist, it might provide a mechanistic basis for the decrease in virulence that is often associated with microbial pathogen adaptation to particular hosts [105,106].

In conclusion, we have shown via gene expression and other methods that, counterintuitively, a generalist parasitoid wasp is better able to suppress the D. melanogaster immune response than a parasitoid wasp that specializes on D. melanogaster and its close relatives. If host hemocytes act as sentinels of infection, hemocyte lysis by L. heterotoma venom may explain the lack of initiation of a regulatory immune response by D. melanogaster. On the other hand, D. melanogaster's response to L. boulardi infection incorporates both the cellular and humoral arms of the immune system, and the Toll pathway appears to be a central regulator of this multi-faceted response. The surprising differences in infection characteristics between these closely related *Drosophila* parasitoids led us to propose that the specialist L. boulardi's more immune evasive infection strategy is adaptive.

## Materials and Methods

### Species strains.

L. boulardi strain Lb17 and L. heterotoma strain Lh14 were generated from single females caught in Wolfskill Orchard, Winters, California, in the fall of 2002. Each strain was inbred by sib-sib mating for at least 12 generations. Ribosomal RNA *ITS2* sequences were collected from each species to ensure species identity [37] and have been deposited under accession numbers DQ218153–DQ218154 in GenBank. Wasp strains will be disseminated upon request to the authors. D. melanogaster strain Ore-R was used for most infection experiments, including those for microarrays. However, in the lamellocyte lysis and encapsulation assays a highly immunocompetent tumor strain *y v hop^Tum-l^* was used. Larvae of this strain show an overabundance of circulating lamellocytes that sometimes encapsulate self tissues to form melanotic tumors.

### Phylogenetic trees.

Wasp ribosomal RNA *ITS2* sequences for all strains besides Lh14 and Lb17 were taken from GenBank [36,37]. Trees were constructed from *ITS2* sequences (gaps included) using the parsimony method as implemented in the program Phylip, version 3.66 (http://evolution.genetics.washington.edu/phylip.html). Bootstrap scores are based on 1,000 resampled datasets.

### Electron microscopy.

Lb17 long glands and reservoirs were prepared for transmission electron microscopy as described [107]. Thin sections (100 nm) were stained with 2% uranyl acetate and viewed in an 80 kV transmission electron microscope (Zeiss EM 902). Images were taken using a MagaView III CCD at a pixel resolution of 1280 × 1040.

### *Drosophila* species infections.

All infections occurred at 22 °C. Either six female Lb17 or five female Lh14 wasps were placed in 35-mm petri dishes filled with 2 ml of fly food and 50 fly larvae (in pilot experiments using D. melanogaster, we found that these wasp/host ratios yielded an approximately equal number of wasp eggs per fly larva). Wasps were removed 2 d later. The proportions of wasps that emerged from each plate were scored and averaged across multiple plates. The average number of flies infected per treatment was approximately 250. While the length of contact between wasps and fly larvae ensures that wasps will have access to host larval stages ideal for successful infection, repeated ovipositions in single fly larvae could result in inflated parasitization success [2]. For wasp/fly species pairs that yielded very few wasp offspring, we did not attempt to distinguish refusal to lay eggs, wasp egg developmental arrest, or host immune response against the eggs as potential reasons for lack of wasp success.

### Staining of melanotic capsules.

Melanized Lb17 eggs were dissected out of D. yakuba larvae 24 h after attack and fixed in 3% paraformaldehyde for 5 min at room temperature. After rinsing in PBS, eggs and associated cells were permeabilized in 0.5% Triton X-100 in PBS for 10 min, and then rinsed again in PBS. Melanized wasp eggs were then stained to determine whether *Drosophila* cells formed a capsule outside the melanized egg. Eggs were stained in 5 μg/ml of the DNA (nuclear) stain Hoechst 33342 for 1 h or in 5 units/ml of the actin (cell membrane) stain rhodamine-phalloidin for one half hour.

### Lamellocyte lysis assay.

We used an in vitro assay for assaying the effects of wasp venom on host lamellocyte morphology [40]. Fifty female wasp long glands and their reservoirs were dissected from both Lb17 and Lh14 and crushed in 50 μl of PBS to obtain wasp venom proteins. Hemocytes from 12 homozygous or hemizygous *hop^Tum-l^* larvae were bled in a spot plate well containing 300 μl of PBS with 7% bovine serum albumin. Seventy μl of medium containing hemocytes was aliquoted into slide wells, and 2.1 μg of wasp protein (Pierce Micro BCA Protein Assay Kit) was added to each well, with straight PBS as control. Samples were kept at 25 °C in gentle rotation, and the proportion of bipolar (lysed) lamellocytes, as described in [45], was recorded at approximately 2 and 4 h after the addition of venom fluid.

### Supernumerary egg encapsulation assay.

Lb17 and Lh14 were allowed to infect homozygous or hemizygous *hop^Tum-l^* larvae, which are stronger melanotic encapsulators than standard D. melanogaster strains. Five days after infection, fly larvae were dissected and the encapsulation of dominant and supernumerary parasite larvae was recorded. Supernumerary wasp eggs/larvae are usually killed by the dominant wasp egg/larva around the time of hatching [2,101].

### Infections for microarrays.

Wasps were allowed to attack late second instar fly larvae (72 h old at 22 °C) in the following manner. Nine petri dishes containing 60 fly larvae were each exposed to six experienced Lb17 female wasps for 2 h, another nine plates were exposed to five Lh14 females, and nine control plates were left uninfected. For each of three time points post-infection (2–5 h, 9–12 h, 21–24 h), 40 larvae from three replicate plates were removed and frozen at −80 °C for RNA extraction and microarray analysis (3 treatments × 3 time points × 3 replicates = 27 samples). To ensure that infectivity of both wasp species was similar under these conditions, 12 of the remaining 20 larvae in each infection plate were dissected and the number of wasp eggs in each larva was scored. The average number of Lb17 eggs across 90 larvae scored was 1.27 (±0.69 SE), while the average number of Lh14 eggs was 1.29 (±0.60 SE).

### RNA extraction and hybridization.

RNA was isolated using the standard Trizol (Gibco) method, and was cleaned using RNeasy spin columns (Qiagen). RNA quality was assayed on an Agilent 2100 Bioanalyzer, and labeled cRNAs were manufactured, fragmented, hybridized to Affymetrix V2.0 *Drosophila* GeneChips, and scanned by the Cornell University Weill Medical College Microarray Core Facility. The expression data reported in this paper have been deposited in the Gene Expression Omnibus (GEO) (http://www.ncbi.nlm.nih.gov/geo/) database under accession number GSE8938.

### Microarray data analysis.

We used GeneTraffic (Stratagene) for microarray analyses. Probe intensities were normalized using Robust Multi-Chip Analysis [108], with each set of nine chips from the three experimental time points analyzed separately, and the three control replicates from each time point designated as baseline. To assess significance of differences in gene expression across treatments, we performed two-class unpaired, unequal variance *t*-tests for every gene. The false discovery rate (or *q*-value) for various probability value cutoffs was estimated in the statistical program R (Table 1) [109]. The program GenMAPP V2.0 was used to assess overrepresentation of significantly differentially regulated genes in particular gene ontology categories (http://www.geneontology.org/) [110].

We chose an initially liberal *t*-test *p*-value cutoff (*p* < 0.01) for identifying significantly differentially expressed genes, which manifested itself in relatively high *q*-values for the groups of significantly differentially expressed genes from different treatment comparisons (Table 1). However, we then limited our focus to the biological function GO categories that were significantly overrepresented by these differentially expressed genes. Because “false positive” genes are expected to be randomly distributed with respect to biological function categories, while “true positive” genes are likely related by biological function, our focus on differentially regulated biological function categories (as opposed to differentially regulated individual genes) likely substantially decreases the number of false positive genes identified with little cost in terms of loss of true positives.

### Phenoloxidase (PO) activity.

We chose to assay PO activity 24 h after infection because this is the time that hosts would normally be producing melanotic capsules around avirulent wasp eggs [8]. Batches of 50 uninfected control, Lb17 attacked, or Lh14 attacked larvae (six replicates of each) were homogenized 24 h post-infection in 50 μl cold 5 mM MOPS buffer. The homogenate was incubated at room temperature for 1 h, and then was aliquoted in 20 μl samples into a 96-well spectrophotometer plate with 130 μl ddH20 and 50 μl of 10 mM L-DOPA. PO activity was measured 1 h later by recording absorbance at 490 nm of the dopachromes formed from the conversion of L-DOPA by PO [111]. Since PO is typically present in host hemolymph as the inactive form proPO, which becomes activated by host proteases or exposure to air, our assay is a measure of the cumulative amount of proPO and PO in the hemolymph samples. A modified Lowry assay (Sigma procedure number P5656) was used to control for the total amount of protein present in each homogenate.

### AMP-GFP fluorescence.

Transgenic D. melanogaster lines with GFP reporter genes cloned downstream of various antimicrobial peptide promoters (*Attacin-A*, *Cecropin A1*, *Diptericin*, *Drosocin*, *Drosomycin*, *Metchnikowin*, [112]) were kindly provided by B. Lemaitre. These lines were assayed for fat body GFP fluorescence after wasp attack. The presence of a single wasp egg was confirmed in each larva prior to observation of its fat body cells. The statistical significance of differences in GFP fluorescence between treatments was calculated using Monte Carlo contingency table tests as implemented in P-Stat (http://engels.genetics.wisc.edu/pstat/index.html). Images were taken using a Zeiss fluorescence microscope at 40× magnification with a two-second exposure.

## Supporting Information

Figure S1 Fat Body AMP-GFP Fluorescence after Wasp Attack (109 KB PDF)

Table S1 Overrepresented GO Categories among Significantly Differentially Regulated Genes (21 KB XLS)

Table S2 Fold Differences between Treatments for Genes from GO Categories Discussed in the Text (A) Fold differences between treatments for genes from gene ontology categories commonly regulated after attack by Lb17 and Lh14.
(B) Fold differences between treatments for genes from gene ontology categories differentially regulated after attack by Lb17 compared to Lh14.
(194 KB XLS)

Table S3 Fold Differences between Treatments for Candidate Anti-Parasite Immune Genes (148 KB XLS)

Table S4 Percent of Genes Differentially Regulated after Attack by Both A. tabida and L. boulardi/L. heterotoma
(16 KB XLS)

Video S1 L. heterotoma Female Ovipositing in D. melanogaster Larva (9.5 MKB AVI)

### Accession Numbers

Ribosomal RNA *ITS2* sequences have been deposited under accession numbers DQ218253–DQ218254 in GenBank (http://www.ncbi.nlm.nih.gov/Genbank/index.html). The expression data reported in this paper have been deposited in the Gene Expression Omnibus (GEO) (http://www.ncbi.nlm.nih.gov/geo/) database under accession number GSE8938.

## Abbreviations

AMP: antimicrobial peptide
GFP: green fluorescent protein
GO: gene ontology
*ITS2*: *internal transcribed spacer 2*
Lb17: *Leptopilina boulardi*, strain 17
Lh14: *Leptopilina heterotoma*, strain 14
PO: phenoloxidase
proPO: prophenoloxidase
VLP: virus-like particle
