# Supplementary material for: Contrasting Infection Strategies in Generalist and Specialist Wasp Parasitoids of Drosophila melanogaster
Source: PLoS Pathog. 2007 Oct 26;3(10):e158. doi: 10.1371/journal.ppat.0030158 (PMC2042021; doi:10.1371/journal.ppat.0030158)

Figure S1. Fat Body AMP-GFP Fluorescence After Wasp Attack

Percent of fat body cells fluorescing after wasp attack for each of six AMP-GFP Drosophila strains: *Attacin-A*, *Cecropin A1*, *Diptericin*, *Drosocin*, *Drosomycin*, and *Metchnikowin*. The Y-axis represents the percent of individual flies in each fluorescence category. P-values for differences between treatments were generated by comparison of 2x5 contingency tables as in Table 2.

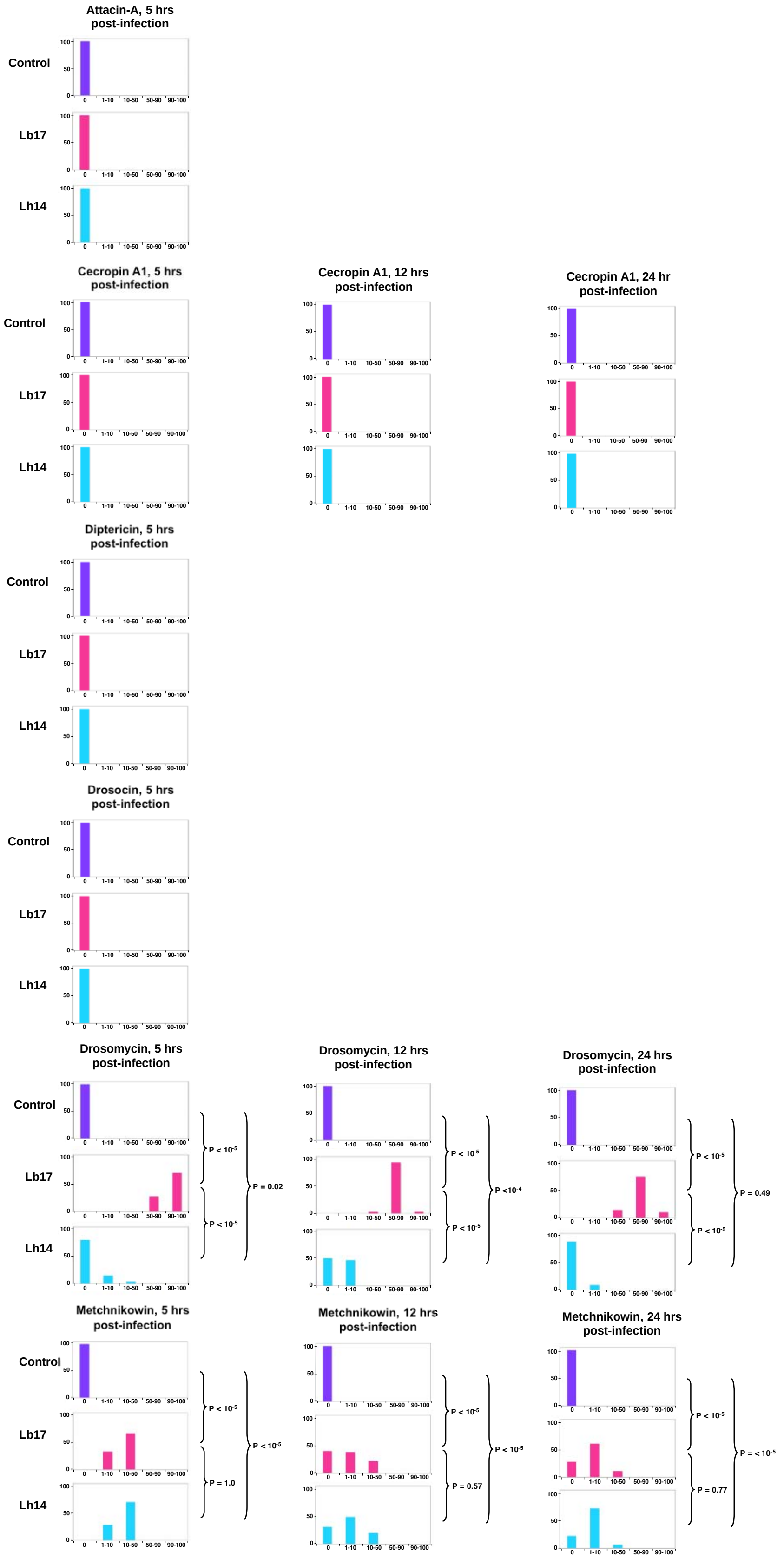

Supplement: Figure S1 — (109 KB PDF) [file ppat.0030158.sg001.pdf]
